# Associations between ethnicity and mental health problems among children and adolescents in the United Kingdom: A systematic review and narrative synthesis

**DOI:** 10.1186/s12889-024-20695-3

**Published:** 2024-11-25

**Authors:** Shengjia Guan, Barry Coughlan, Kate Evans, Robbie Duschinsky

**Affiliations:** Primary Care Unit, Department of Primary Care and Public Health, University of Cambridge, Forvie Site, Robinson Way, CB2 0SR Cambridge, United Kingdom

**Keywords:** Mental health, Mental health problems, Ethnic minority, Children, Adolescents, Narrative synthesis, Ethnic disparities

## Abstract

**Background:**

The associations between ethnicity and mental health problems (MHPs) among children and adolescents in the UK have been reported in recent years. However, this is the first review to compare and synthesise these associations and provides a deep understanding of child MHPs across ethnic groups in the UK.

**Method:**

A comprehensive literature search across seven electronic databases and fifteen websites was conducted. The inclusion criteria focused on studies reporting quantitative associations between ethnicity and MHPs for children and adolescents aged 0–19 residing in the UK. Given the high heterogeneity of the studies, a narrative synthesis was adopted to analyse the associations.

**Results:**

Twelve studies met the inclusion criteria, involving a total of 48,281 participants. The review reports no significant differences in the risk of experiencing general MHPs among children from Indian, Pakistani, Bangladeshi, and Black Caribbean groups compared to their White British counterparts. However, Black African children were less likely to develop general MHPs, while children in the Pakistani, Bangladeshi, and Black Caribbean groups showed a higher risk for internalising problems. Externalising and conduct problems were similarly likely among children from Pakistani and Bangladeshi backgrounds compared to White children, with Black Caribbean children showing a higher likelihood of these issues.

**Conclusion:**

The findings suggest that most ethnic minority children and adolescents in the UK have comparable risks of MHPs to their White counterparts, although specific risks vary by ethnicity and MHP types. The results underscore the need for multifaceted analyses considering socioeconomic and cultural factors, beyond simple ethnic categorisations, to inform mental health services that effectively meet the diverse needs of the UK’s child population. This review calls for more detailed and uniform categorisation in future research to understand and address the mental health disparities across different ethnic groups.

**Supplementary Information:**

The online version contains supplementary material available at 10.1186/s12889-024-20695-3.

## Background

With the increasing diversity of the UK population [1], some research suggests that the mental health of children and adolescents from ethnic minority groups may be shaped by complex and multifaceted factors, leading to varied associations between ethnicity and mental health problems (MHPs). Studies by Zilanawala et al. [2] and Platt [3] suggested that children of Pakistani families are at a higher risk of experiencing mental health difficulties compared to White peers, while Amhad et al. [4] reported a lower risk. In addition, the influence of potential confounding factors on the associations appears to be complicated. Midouhas [5] reported that children of Bangladeshi communities are less likely to suffer from externalising problems when adjusted for pupil-level free school meals (FSMs), child characteristics, and family-related factors. Conversely, Karamanos et al. [6] reported a higher likelihood of experiencing these problems with increasing PM2.5 pollution levels. These intricate associations underscore the significance of understanding nuanced ethnic differences to effectively monitor mental health inequalities. Given the complex and conflicting associations presented in the studies above, this review seeks to determine whether these findings are consistently reflected across the wider body of literature.

However, efforts to synthesise the existing evidence and identify overarching patterns are made challenging due to the equivocal and fragmented nature of the evidence. Firstly, MHPs are diverse. Some studies have used broad and umbrella terms such as ‘mental illness’ and ‘mental health difficulties’ to describe general MHPs [7, 8], while others have focused on more specific mental issues, such as externalising problems, internalising problems, self-harm, and psychosis [7, 9–12]. Secondly, the categorisation of targeted ethnic groups is inconsistent across studies. Some researchers have used dichotomies to divide the general population into two ethnic groups: White British and ethnic minority groups [11, 13]. Other research, however, has investigated child mental health across multiple ethnic groups within a single study and used varying levels of ethnic categorisation. For example, Patalay and Fitzsimons [7] used five high-level ethnic categories: White, Asian, Black, Mixed, and Other. Astell-Burt et al. [14] offered six more detailed ethnic groupings in their analysis: White British, Indian, Pakistani and Bangladeshi, Black Caribbean, Nigerian and Ghanaian, and Other African.

The fragmented state of the literature has been further highlighted by the limited scope of systematic reviews in this area. This review focuses exclusively on studies conducted in the UK due to the UK’s specific ethnic distribution [1] and its unique healthcare system [15], which differ significantly from other countries such as the US [16] and Australia [17]. As a result, extending the scope to international studies would not provide applicable evidence for this review. So far, the systematic review by Goodman et al. [18] in 2008 is the only work to date that investigates child mental health differences among ethnic groups in the UK. The review synthesised semi-structured descriptive results from relevant studies spanning 1972 to 2007. The authors concluded that children from ethnic minorities tended to show a comparable or lower prevalence of MHPs relating to internalising and externalising problems compared with White British children but a higher prevalence for certain specific disorders such as self-harm.

Over a decade has passed since Goodman et al.’s review, and with new research on the topic emerging, there is a need for a new systematic review to synthesise the current evidence and address the identified research gaps. Such a review is essential for researchers, policymakers, and practitioners to understand the complex links between ethnicity and child MHPs in the UK.

This systematic review is motivated by the following research question: what are the associations between ethnicity and MHPs among children and adolescents in the UK? If the review reveals significant associations between certain ethnic minority groups and mental health issues, it could inform targeted services and thus promote equitable mental health outcomes across ethnicities. Conversely, a finding of few or weak associations would suggest that universal, rather than ethnicity-specific, mental health services are sufficient to meet the needs of British children. Thus, the findings of this review have the potential to directly influence policy and healthcare provision strategies.

## Method

### Search strategy

We conducted a comprehensive literature search in line with the Preferred Reporting Items for Systematic Reviews and Meta-Analysis (PRISMA) guidelines [19]. Searches of Title/Abstract and subject index headings were run in seven electronic databases (including MEDLINE via Ovid, APA PsycINFO via ProQuest, Embase, PubMed, Web of Science, Scopus, and Applied Social Sciences Index and Abstracts (ASSIA) via ProQuest) and fifteen websites (see Additional file 1). Terms related to (a) population of interested age groups (e.g. “child*” and “adolescen*”), (b) population of interest by ethnicity (e.g. “ethnic*”, “race”, and “immigra*”), (c) location of interest (e.g. “UK”, “Britain”, and “England”), (d) mental health outcomes (e.g. “mental health”, “internalising problems”, and “PTSD”), and (e) mental health services (e.g. “CAMHS” and “mental services”) were combined (see Additional file 1). Searches were conducted in November 2022. Large epidemiological population-based studies and surveys, which might include relevant information, were also searched. Backward citation tracking (i.e. identifying studies that cited articles of interest) and forward citation tracking (i.e. scanning reference lists of included studies) were also conducted to include potentially eligible literature.

### Inclusion and exclusion criteria

Studies were considered eligible for inclusion if:

1. they reported quantitative data on mental health outcomes (i.e. effect sizes of the associations), and results were related to participants’ mental health, either in umbrella terms (e.g. “psychological well-being” and “mental health difficulties”) or in specified diagnoses (e.g. conduct problems, behavioural disorders, psychosis, eating disorders, suicide, and self-harm);
2. their samples contained children and/or adolescents aged 0–19 years residing in the UK. The upper age limit aligns with the definition provided by the World Health Organization (WHO) [20] and the UK’s National Health Service (NHS) [21]. This age range is consistent with a previous systematic review focusing on children’s mental health in the UK [18];
3. at least two categories of ethnic groups were included, with one being White or White British. This unified approach allows for synthesising data from multiple studies to compare the outcomes of each minority ethnic group with those of the White/White British population;
4. mental health conditions were measured using validated tools (e.g. the Strengths and Difficulties Questionnaire [SDQ], a structured 25-item questionnaire used to screen for symptoms of childhood emotional and behavioural psychopathology [22]), clinical interviews or questionnaires, or by referrals to or admissions into child mental health services (e.g. CAMHS).

Studies were excluded if they:

1. exclusively targeted the adult population or did not present subgroup analysis results for participants aged 19 and under;
2. focused exclusively on selected at-risk groups of children and/or adolescents, such as homeless youth or those in child protective services custody, juvenile detention facilities, or foster care; this criterion is applied because the goal of the review is to provide a broader and more representative understanding of the general UK population;
3. Were case studies, commentaries, or editorials
4. involved participants with a primary diagnosis of physical illnesses – such as cystic fibrosis or cancer – other than MHPs;
5. targeted a population with pervasive or circumscribed developmental disorders, such as autism, language development disorders, or an intelligence disability.

### Study screening

**Fig. 1:**
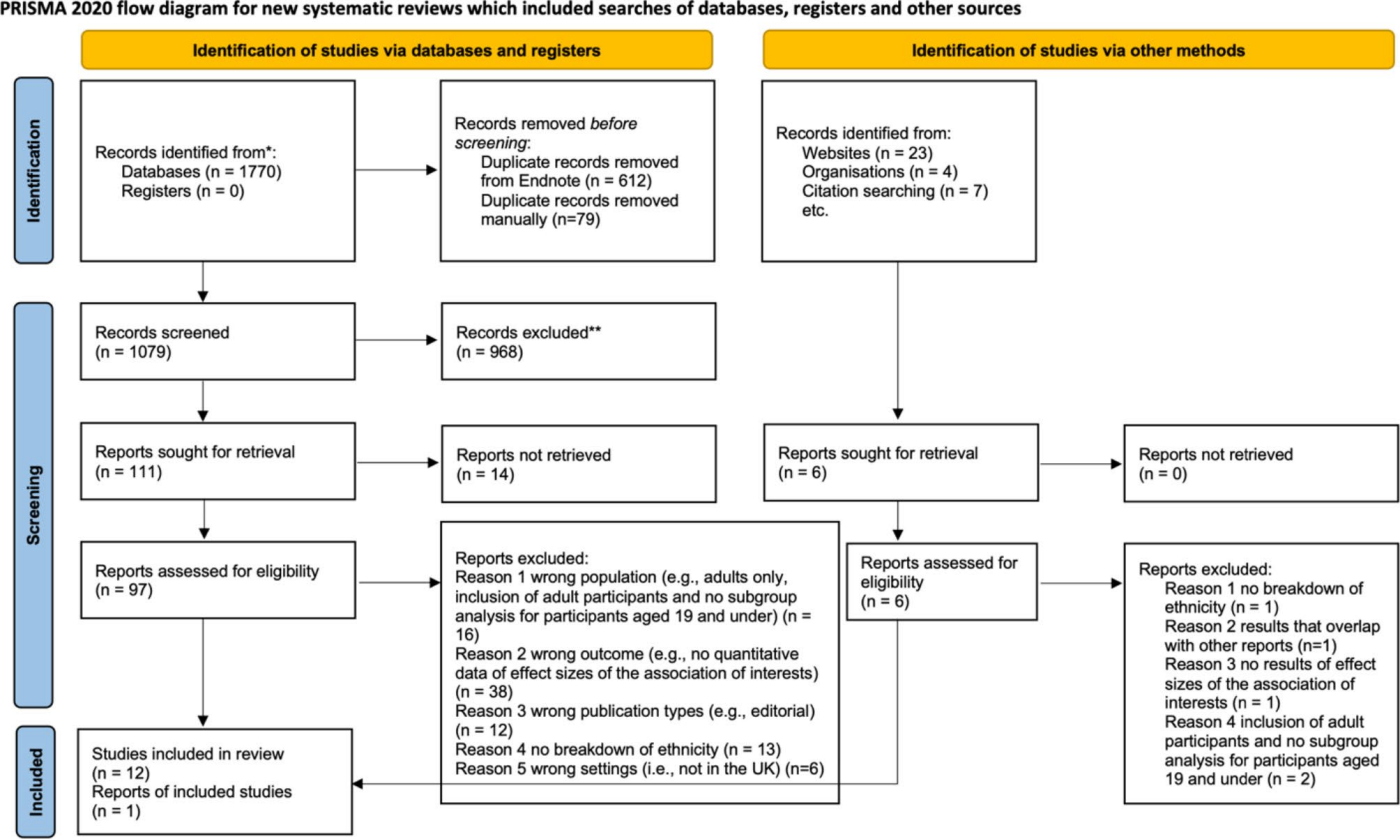
PRISMA 2020 flow diagram for the systematic review

A total of 1804 citations were initially searched across the aforementioned databases and websites. Additionally, the reference lists of included articles and literature citing included articles were manually searched for additional relevant publications. Of these, 691 duplicates were removed using EndNote 20 and through manual comparisons. Two reviewers (SG and KE) screened the titles and abstracts of the first 200 articles together. The remaining 879 citations were screened independently. Disagreements regarding five articles were resolved through discussion and consultation with RD and BC. Overall, 12 records were deemed eligible for inclusion in this review (see Fig. 1 for the PRISMA flow diagram).

### Data extraction

Data extraction was performed by SG and KE using a pre-determined data extraction table (see Additional file 2 for more details). These authors collaboratively extracted data from two randomly selected studies, resolving discrepancies through conversation before independently extracting data from the rest of the studies. The information extracted involved study characteristics (e.g. study design, participants’ demographics, data collection/source, ethnicity assignment, mental health outcome(s), outcome measure(s), analysis method, and results of interest). The methodological limitations listed in the Additional file 2 were derived from a previous systematic review on a similar topic by Goodman et al. [18]. Discrepancies in data extraction were resolved through discussion with RD and BC to achieve consensus.

**Table 1:** Quality assessment results

	Section A: Are the results of the study valid?						Section B: what are the results			Section C: Will the results help locally?			
**Study Ref.**	**Q1**	**Q2**	**Q3**	**Q4**	**Q5a**	**Q5b**	**Q7**	**Q8**	**Q9**	**Q10**	**Q11**	**Q12**	**Overall quality**
Bhui et al. [23]	H	H	H	H	H	H	H	H	H	M	H	H	H
Platt [3]	H	H	H	H	M	H	H	H	H	M	M	M	M
Vostanis et al. [13]	M	H	L	M	L	L	M	L	L	L	M	M	L
Singh et al. [24]	H	H	H	H	H	H	H	H	H	M	H	H	H
Zilanawala et al. [2]	H	H	H	H	H	H	H	M	H	H	H	H	H
Patalay et al. [7]	H	H	H	H	H	H	H	H	H	H	H	H	H
Midouhas [5]	H	H	H	H	H	H	H	L	M	H	M	H	H
Jonsson et al. [8]	H	H	M	M	H	H	H	M	M	M	H	M	M
Karamanos et al. [6]	H	H	H	H	H	H	H	M	M	M	H	M	M
Blakey et al. [9]	H	H	H	H	H	H	H	H	H	M	H	M	H
Farooq et al. [12]	H	M	H	H	M	H	H	H	M	L	H	H	M
Ahmad et al. [4]	H	H	H	H	H	H	H	M	M	H	H	H	H

**Note**: ‘H’ refers to ‘High quality’, ‘M’ is ‘Moderate quality’, and ‘L’ is ‘Low quality’.

### Quality assessment

The Critical Appraisal Skills Programme (CASP) cohort study checklist [23] was used to appraise the quality of the studies included in this review. This checklist provides 12 questions to assess study quality (see Additional file 3 for the checklist template). The first three items serve as screening questions, while the remaining nine offer a framework for assessing the study’s results, validity, and relevance. Due to this review’s primary focus on exploring the associations between ethnicity and MHPs, question 6, ‘*Was the follow-up of subjects (a) complete enough and (b) long enough?*’ was removed from the checklist. The CASP checklist does not provide a scoring system to appraise the quality of evidence. Qualitative quality rating criteria were established by SG (see Additional file 4 in appendices) based on hints provided in the checklists and the specific research question of this review. RD reviewed the criteria regarding their suitability and feasibility.

The quality assessment was independently conducted by SG and subsequently reviewed by RD; uncertainties were resolved through discussion. It is important to note that the CASP assessment was not used to exclude or weight any of the included studies; however, the assessment results (see Table 1) informed the data synthesis and the interpretation of findings.

### Data synthesis

High heterogeneity was observed across the studies, including various categories of MHPs, different assignments for ethnic groups, inconsistent measures of MHPs, different age ranges of the population, different types of models (i.e. base models and adjusted models) in statistical analysis, and different confounds across models. Such heterogeneity was not intended as a quantitative measure of effect size variability but rather as a qualitative judgement of the methodological heterogeneity present in the included studies. Therefore, it was challenging to aggregate the studies’ data into a single quantitative scale for pooling and thus a narrative synthesis approach was adopted over a meta-analysis.

The synthesis steps were developed based on guidance for narrative synthesis in systematic reviews by Popay et al. [24], integrating into this review’s aim:

- summarise key characteristics of each included study (including authors and years, study design, settings, targeted population with age range, MHPs, measurement tools, type of statistical analysis models, and main findings);
- divide the included studies into various types of MHPs, including an umbrella term of general MHPs (GMHPs) and other specific types of mental problems; this approach helps identify patterns or differences across studies in the kind of mental health needs considered;
- assess the quality of included studies through the quality assessment and other factors that might influence results, such as sample size, and potential biases during the review process;
- identify patterns, trends, and contrasts between studies.
- identify factors that could explain differences and contrasts in findings between studies, such as study quality differences, methodological differences, and data source (e.g. sample sizes, settings, and population and context characteristics);
- assess the robustness of the synthesis by analysing the validity of measures for MHPs and ethnicity, the quality of the included studies, and any potential biases during the review process.

The effect sizes of the associations are presented in Additional file 5 (see Appendices) to provide an overview of the associations between ethnicity and MHPs in the UK. Results from both baseline and adjusted models were synthesised.

## Results

### Overview of included studies

From 1,804 identified citations, 12 met the eligibility criteria for analysis, involving participants aged from 9 months to over 19 years, with the majority (k = 7/12) focusing on ages 10–16 [4, 6–9, 13, 25]. The sample sizes varied from 2,900 to 12,376, with a grand total of 48,281 participants. To avoid duplication, only the largest participant count was included for studies using the same cohort (e.g. the Millennium Cohort Study [MCS] [26]). Among the 12 studies, 11 were population-based, and one was clinic-based, with quality assessments showing seven as high-quality (58.3%), four as moderate (33.3%), and one (8.3%) as of low quality.

All studies included White or White British groups as a reference for analysis, with 30% using high-level ethnicity categories (e.g. White, Asian, Black) and the rest focusing on specific ethnicities (e.g. Indian, Bangladeshi, Pakistani). Ethnicity assignment varied, with four studies [2, 3, 5, 7] using caregiver reports based on UK census [27] categories, three utilising self-reported ethnicity [4, 8, 9], and two combining self-report with parental and grandparental birth countries [6, 25].

Regarding MHPs, six studies used umbrella terms such as ‘mental health difficulties’, two [2, 5] focused on internalising and externalising problems, two [6, 9] on conduct problems, one [28] on psychosis-like symptoms, and one [12] on self-harm. Data collection methods also varied: one study [13] collected data directly from schools with an over-representation of children with Indian backgrounds, while the others used established datasets such as the MCS [26], the Determinants of Adolescent Social well-being and Health (DASH) study [29], and the Ethnicity and Adolescent Mental Health (REACH) study [30]. Study designs included six longitudinal, five cross-sectional, and one observational cohort study, predominantly representing populations from England, especially London (k = 4/12, 33.3%).

For the measurement of MHPs, six studies used self-reports, four used parent reports, one employed trained interviewers, and one relied on specialist assessment. The SDQ was the most common measure [22], used in 10 studies via self-reporting (k = 6/10) and parent-reporting (k = 4/10). The SDQ has been translated into more than 80 languages [31]. The average SDQ scores can calculate the estimated prevalence within sub-groups delineated by various characteristics, such as ethnicity, in the UK [32]. Other measures included the Psychosis-like Symptoms Interview (PLIKSi) [28] and a specialist psychosocial assessment for self-harm cases [12].

The number of publications on ethnic minority children’s mental health has been low over the past decade, with a slight increase in 2021, possibly related to increased attention to matters of ethnicity following the George Floyd protests in May 2020 [33].

### Narrative synthesis

The synthesis analysed effect sizes along with their 95% confidence intervals (CIs) or stand errors (SEs) from the included studies (see Additional file 5 in Appendices), categorising types of MHPs and ethnic groups for direct comparison within mental health categories. Both base and adjusted models (e.g. controlling age, gender, socioeconomic status, and deprivation index) were considered, revealing varying risks of MHPs across ethnicities. Effect sizes were reported as regression coefficients, odds ratios (ORs), risk ratios (RRs), hazard ratios, and fixed effects estimates. These metrics quantify the strength of the associations.

Most models did not find significant differences at the 5% significance level (i.e. *p* ≥ 0.05 and/or having a 95% CI that includes the null value) between ethnic minority groups and the White/White British group. However, this synthesis does not rely solely on p-values. CIs have also been considered as they provide a more informative and convincing approach [34]. It also provides a more comprehensive view of the effect sizes and their direction, magnitude, and precision.

### General MHPs (GMHPs)

GMHPs refer to the problems that were described using umbrella terms, such as ‘mental illness’ and ‘mental health difficulties’, rather than specific types of mental problems. The majority of included GMHPs were measured based on the total difficulties score (TDS) of the SDQ, including scores summed by emotional symptoms (five items), conduct problems (five items), hyperactivity/inattention (five items), and peer associations problems (five items) [22].

Children and young people in the Black African ethnic group were less likely to develop GMHPs in most included models (k = 4/7, corresponding to 4 out of 7 reported effect sizes from base and adjusted models shown in the Additional file 5), compared to their White counterparts. In Amhad et al.’s study [4], the results were stratified by sex, and only boys of Black African communities who were 14 years old were reported with a lower risk than their White British peers in base and adjusted models while controlling for social support, participation (e.g. socialising, religious attendance), and adversity. Despite this reported lower risk, the 95% CIs or standard errors (SEs) were relatively wide or large (see Additional file 5), indicating low precision and uncertainty over the effect sizes. Unlike the boys, no association was found among girls in the models (i.e. p-values were larger than 0.05, and the corresponding 95% CIs were wide and contained the null value of 1).

Our results also show that children in the Indian (k = 7/12), Pakistani (k = 8/11), Bangladeshi (k = 9/11), and Black Caribbean (k = 9/11) groups did not differ significantly in terms of experiencing GMHPS compared to their White counterparts (p-values in most the models were not statistically significant and the corresponding 95% CIs contained the null value of 1). Although some significant results (k = 5/12) existed for the children of Indian families, the precision of the results was relatively low. These still indicate a lack of significant results, which indicates that there might be no association between children with Indian background and GMHPs in the UK.

Other ethnic groups – such as male children of Nigerian/Ghanaian, Asian, and Black groups – reported a lower risk of suffering from GMHPs. However, conclusions cannot be drawn due to the limited number of results.

### Externalising problems and conduct problems

In the four studies that examined externalising or conduct problems [2, 5, 6, 9], most models did not identify statistically significant associations between children of Indian (k = 6/8), Pakistani (k = 6/8), or Bangladeshi (k = 5/8) groups and the prevalence of externalising or conduct problems.

Black Caribbean children and young people (k = 5/10, 50%) might be more likely to experience GMHPs compared to their White counterparts. Although only half of the included effect sizes reported such associations, their corresponding SEs or 95% CIs indicated the results had high precision, increasing our confidence in the findings. However, it is important to note that most significant results were from the models with fewer controlled variables [2, 6]. The wide range of 95% CIs or large SEs of the non-significant results from the adjusted models might imply increased uncertainties because of too many confounding variables, especially if some confounds are not genuinely relevant to the outcome.

Contrasting results were found in studies with young people in the Black African ethnic group compared to those in the White ethnic group. Zilanawala et al. [2] and Midouhas [5] reported that children of Black African families were less likely to have conduct problems than White British children with relatively high precision for the effect sizes (i.e. small SEs). In contrast, Karamanos et al. [6] reported positive associations between children of Black African groups and conduct problems in an adjusted model considering air pollution (i.e. NO_2_ levels) and in a model adjusting for more confounds in the context of PM2.5 exposure. The contrasting findings in these studies might be attributed to their different focuses, methodologies, and study quality. Karamanos et al.’s study (moderate quality) considered the interactive effects of air pollutants and ethnicity on child conduct problems, while Zilanawala et al. (high-quality study) examined the direct relationship between ethnicity and externalising problems and Midouhas (high-quality study) explored the impact of school poverty on behavioural trajectories, considering the role of ethnicity. These varying approaches might highlight how environmental, socio-economic, and direct ethnic factors can influence the outcomes differently.

Drawing conclusions regarding the ‘Other’ ethnic category is challenging due to the varying definitions used in the studies. It is difficult to draw definitive conclusions for certain ethnic groups, including ‘Other Whites’, ‘Any other mixed’, ‘any other Black’, ‘Latin American’, and ‘Mixed White and Black’, as only one study was conducted on each of these groups.

### Internalising problems

Two studies [2, 5] examined internalising problems. Children of Indian (k = 3/4, 75%) and Black African (k = 2/4, 50%) ethnic groups had a similar risk of internalising problems compared to White peers. However, Zilanawala et al.’s study indicated that children in the Black African ethnic group were significantly more likely to experience MHPs compared to those in the White ethnic group (*p* < 0.05), from the result of the adjusted model (coefficient = − 0.16, SE = 0.19) although the large SE relatively to the coefficient implies considerable uncertainty and might suggest no effect.

In addition, all models showed that children and young people in the Pakistani ethnic group had a higher risk of experiencing mental health disorders compared to their white peers. Similar results were also found with children in Bangladeshi and Black Caribbean ethnic groups by Zilanawala et al. [2], while Midouhas reported no association [5]. Compared to Midouhas’s study, Zilanawala et al.’s had a larger sample size, a larger effect size with higher levels of precision and less uncertainty of the results (i.e. smaller SEs), and a more direct focus on the relationships between ethnicity and internalising problems. These reduce the chance of type 1 and type 2 errors and increase the reliability and likelihood of true positive associations.

### Psychosis-like symptoms

One study focused on psychosis-like symptoms. Singh et al. assessed the association between definite psychotic-like symptoms (PLIKS) and ethnicity among 1–12-year-old children [28]. Their findings indicated no statistically significant association between PLIKS and non-White children compared to their White peers, with an odds ratio (OR) of 1.50 (95% CI: 0.93–2.43) in the unadjusted model and an OR of 1.05 (95% CI: 0.56–1.97) after adjustments for urbanity, family adversity, residential and school mobility, and peer difficulties. The outcomes were measured using a valid face-to-face, semi-structured PLIKS interview (PLIKSi) derived from the Diagnostic Interview Schedule for Children, version IV (DISC-IV) [35] and the Schedules for Clinical Assessment in Neuropsychiatry version 2.0 (SCAN) [36]. Nevertheless, drawing definitive conclusions from this single study is challenging due to its solitary nature and geographical limitations (data collection was confined to Avon rather than nationwide).

### Self-harm

Farooq et al. [12] targeted self-harm and explored its hazard ratios of repeat self-harm in children and adolescents with a complete 12-month follow-up for 10–19-year-old adolescents of ethnic minority groups compared to their White counterparts. The study suggested that Black adolescents exhibited a reduced hazard of repeated self-harm in both unadjusted and adjusted models, the latter accounting for age, sex, level of deprivation, previous self-harm incidents, and the method of self-harm. Additionally, South Asian and other non-White groups demonstrated a lower risk with narrow CIs, indicating a high level of reliability in the effect sizes reported, although the statistical significance of these findings was confined to the unadjusted models. Despite the precision of these findings and the considerable sample size (11,906 adolescents), the generalisability of the results to the broader UK context is restricted, as the data were derived from clinical settings.

## Discussion

### Comparison with previous review

This review’s examination of the risk of MHPs across ethnicities provides the prevalence of MHPs in children and young people, which offers a different methodological approach to Goodman and colleagues’ 2008 study [18]. While the earlier review provided prevalence rates derived from age and gender-controlled models, the current analysis expands the scope by utilising both unadjusted and adjusted models. In particular, adjusted models incorporate a broader range of potential confounding variables, including socioeconomic status and maternal mental health, to accurately measure complex associations involving covariates that impact child mental health outcomes in the real world. Despite the challenges in directly comparing this review’s results with those of Goodman et al., due to their different focuses, they both identify similar patterns: ethnic minority children and adolescents in the UK generally show comparable or sometimes lower prevalence or risk of common MHPs than White British children. By integrating additional confounds, this review not only supports some of Goodman et al.’s findings but also highlights issues of intersectionality, highlighting the importance of conducting multifaceted analyses.

### Overarching trends of the associations

This systematic review focuses on the relationship between ethnicity and child MHPs in the UK, incorporating data from 12 studies. Given the diverse nature of the studies, a narrative synthesis was chosen over a meta-analysis to assess the associations.

Overall, this review reveals that most ethnic minority children and adolescents have a comparable risk of experiencing MHPs as their White counterparts, although inter-ethnic and inter-study disparities do exist. Some studies, mentioned in the Background, point to striking ethnic disparities in child mental health outcomes. However, synthesising a range of studies, these results were not found consistently. A review of these studies suggests that associations between ethnicity and mental health are shaped by multiple factors, and ethnic minority status alone may not consistently result in higher or lower risks of MHPs.

Specifically, the review reveals that there were no significant differences in experiencing MHPs between children in the Indian and White ethnic groups. However, the risk of experiencing MHPs among other ethnic groups varies depending on the types of problems involved. For GMHPs, there were no significant differences between children in the Pakistani, Bangladeshi, and Black Caribbean groups compared to White peers. In contrast, Black African children were reported to be less likely to develop GMHPs. For internalising problems, the risk for children in the Black African ethnic group aligns with that of White children. However, children in the Pakistani, Bangladeshi, and Black Caribbean groups were reported to be more likely to face these issues.

In terms of externalising and conduct problems, children of Pakistani and Bangladeshi groups appear to have similar risks to those in the White ethnic group. Those in the Black Caribbean ethnic group are more likely to experience externalising and conduct problems compared to White individuals, especially in base model analyses. These findings suggest the need for consideration of additional variables, as base models may not fully capture the influence of potential confounding factors such as socioeconomic status. Goff et al. [37] also highlighted that such findings need to be interpreted cautiously and with an understanding of societal biases since Black children, particularly boys, are often perceived as less innocent and more mature than their White peers, which leads to harsher treatment and less protection [37]. Such dehumanising perceptions can result in biased reporting and interpretation of conduct problems, underscoring the need for careful consideration of how racial stereotypes impact our understanding of behavioural issues across different ethnic groups.

This review also shows that the overall likelihood of developing psychosis-like symptoms is comparable between children in White and non-White ethnic groups. Additionally, Black, South Asian, and other non-White children are less prone to self-harm than White children. Nevertheless, these differences were most apparent in basic models and no ethnic variations were reported in most adjusted models. This pattern suggests that factors beyond ethnicity, such as level of deprivation [12], may play a crucial role in understanding the true associations between ethnicity, self-harm, and psychosis. It also highlights the importance of a multifaceted approach in mental health research, where simple ethnic categorisations are insufficient to reflect complex mental health dynamics in their social and economic context.

The review also highlights challenges in using broad ethnic categories such as ‘non-White’, ‘Other’, and ‘ethnic minority groups’. The inconsistent definitions of these groups across studies lead to challenges in accurately generalising findings. This inconsistency might also cause oversimplification, potentially masking the unique risks of having MHPs within specific ethnic subgroups. Furthermore, it indicates a need for more detailed and uniform categorisation in future research.

### Equivocal findings

The diverse findings across the included studies in this review and the equivocal reasons behind these variations present a complex landscape for analysis. First, ethnicity seems to have an important role in the MHPs of children and young people. The results show that children and adolescents from different ethnic minority backgrounds might be exposed to different types of mental health risks, although the underlying reasons are not fully clear. For example, children from Pakistani and Bangladeshi backgrounds showed similar risks for GMHPs and externalising problems as their White peers, yet they also had a higher risk of internalising problems. Such variation was evident despite the studies being of comparable quality and having comparable sample sizes and community-based settings. This observation suggests that such methodological factors might not fully elucidate the reasons behind the variations. A potential explanation is related to the South Asian culture, including that of the Pakistani and Bangladeshi communities, in which emotional distress might be more internalised due to cultural norms around emotional self-control (i.e. behaving appropriately rather than acting on how one is feeling) and collectivism (i.e. placing group welfare before individual welfare) [38]. Such cultural values might result in higher risks of developing internalising problems instead of externalising problems. This complexity in findings underscores the need for future research to explore cultural and contextual factors that might influence the varied mental health risk profiles within the same ethnic minority groups.

In addition, the observed inter-ethnic differences may be attributable to potential confounding factors. Factors such as socioeconomic status, maternal mental health, and experiences of racism were considered in the adjusted models, resulting in most effect sizes becoming reduced or statistically non-significant. This suggests that the ethnic disparities in MHPs reflect these external factors more than ethnicity itself. However, a limitation arises in the way most studies handle confounding variables. In their analysis, Zilanawala et al. used several statistical models to separately adjust for various factors, including cultural traditions, socioeconomic position, perceived racism, maternal psychological distress, and family environment markers, with a final model combining all covariates [2]. In contrast, most studies controlled for multiple confounds collectively in a single model, which makes it challenging to identify the specific impact of each factor on inter-ethnic risk differences. Therefore, future research might consider analysing these confounding factors separately in distinct models, allowing for a more precise assessment of the individual contributions of each factor to the ethnic differences observed in MHPs.

### Robustness of the synthesis

This review raises considerations regarding the validity of mental health measures used in their populations of interest. Although the included studies predominantly utilised the SDQ as a mental health assessment tool (k = 10/12), the specific validity of this measure for certain ethnic minority groups in the UK remains unclear. Despite being one of the most widely and internally used measures of child and adolescent mental health [22], the SDQ may not account for the unique cultural contexts and perceptions of mental health across different ethnicities. For example, the stigma associated with mental illness in South Asian communities could lead to underreporting in parent-reported measures [39]. Furthermore, none of the studies that used the SDQ used the home language versions of the SDQ for populations with limited English proficiency, potentially overlooking idiomatic expressions of mental distress specific to these groups [40, 41]. Such oversight raises questions about the SDQ’s robustness in capturing the true risk of having MHPs among diverse ethnic minority children. Additionally, the validity of other assessment methods, such as the PLIKSi used by Singh et al. [28] and the specialist assessments used by Farooq et al. [12], might be compromised by unknown racial biases or stereotypes held by the interviewers or clinicians [42]. Future research should, therefore, prioritise exploring the cultural adaptation and validity of these mental health assessment tools across different ethnicities within the same nation.

It is also crucial to consider the measurement quality and implications of how ethnicity is measured in the included studies. The measures of ethnicity in the reviewed studies were generally of high quality, typically based on validated methods such as the UK census and parent- or self-reported ethnicity. However, the use of broad ethnic categories (e.g. White, Asian, Black, and ‘Other’) may limit the internal heterogeneity within these ethnic minority groups. The inconsistent definition of some terms (e.g. ‘White’, ‘Other’, ‘non-White’, and ‘Other White’) also adversely impacts the robustness of the synthesis. These features could limit the ability to draw specific conclusions about the data.

In most included studies (k = 10/12), the proportion of ethnic minority children was over-represented to ensure a meaningful analysis of these groups. Despite this, the sample sizes of ethnic minority children were often too small compared to their White or White British counterparts, affecting the statistical power of these studies. The reduced power subsequently increases the chance of Type II (false negative) errors – where the true risk differences between ethnic minority groups might be undetected [43] – potentially limiting their ability to detect differences in mental health outcomes across groups. As a result, the observed ethnic comparisons need to be interpreted cautiously, and limitations of statistical power imposed by the small sample size of ethnic minority children should be acknowledged.

In addition to addressing the potential for Type II errors, we carefully consider the impact of Type I (false positive) errors in interpreting its findings. A standard significance level of *p* < 0.05 was selected to reduce the likelihood of these errors. Despite this, there remains a 5% probability of having Type I errors even with statistically significant results [34]. Consequently, the review has adopted a cautious approach in interpreting findings with p-values. Existing research suggested that integrating p-values with CIs creates a more nuanced interpretation of statistical significance, moving beyond strict cut-off points. This is partly because CIs assess the precision of effect sizes, thus enhancing the robustness of statistical conclusions [34, 43]. This combination of p-values and CIs in our statistical approach has helped interpret findings while acknowledging the trade-off between Type I and Type II errors.

The generalisability of the findings from this review is moderate, slightly impacting the overall robustness of the synthesis. Five of the twelve studies analysed data from the MCS, encompassing rural and urban populations across various UK regions and offering a relatively broad demographic scope. However, the remaining studies predominantly focused on specific areas, especially London. Considering that London is the UK’s most ethnically diverse region [44], these studies might not accurately represent the MHPs of children from less ethnically diverse areas. This geographical focus potentially limits the applicability of the findings across the entire UK, thus affecting the overall robustness of the synthesis.

Additionally, the findings of this review may not be generalisable to other countries due to differences in ethnic distributions and healthcare systems. For example, the UK’s ethnic minority populations are predominantly from South Asian, Black African, and Caribbean backgrounds, reflecting historical migration patterns from Commonwealth countries [1]. In contrast, in the US, the largest ethnic minority group is Hispanic and Latino, which introduces different socio-cultural factors affecting mental health outcomes [16]. These variations in ethnic group distribution result in distinct challenges and protective factors for children in each country. Thus, generalising the findings from this review to other international settings should be cautiously approached.

Overall, robustness has been evaluated by thoroughly analysing the methods used in the included studies. Concerns about the SDQ’s validity across different ethnic contexts due to cultural and linguistic factors affect its reliability. The small sample sizes for ethnic minority children increase the risk of Type II errors, which impacts the detection of actual ethnic differences. While using both significance levels and CIs strengthens credibility, the focus on urban areas – particularly London – limits the study’s generalisability across the UK. Future research should prioritise culturally adaptable psychological measurement tools and include larger, geographically diverse samples.

### Strengths and limitations

This review has three main strengths. First, it synthesises findings on child mental health across ethnicities in the past ten years and is the only review conducted since 2008 [18]. Second, it considers not only base models with or without adjustment for age and gender but also models controlling for potential confounding factors. Such a method enables a better understanding of how various factors might influence associations, revealing real-world associations and potential causes of inter-ethnic differences. For instance, children of Pakistani groups had a higher risk of externalising problems than White British children in the base model adjusted for age and gender. However, the risk between the two ethnicities became similar in the model adjusted for socioeconomic position [2], indicating that the ethnic disparities initially attributed to ethnicity, age, and gender are more influenced by socioeconomic conditions. Third, the review enhances statistical interpretation by using CIs alongside p-values, thereby avoiding simplistic binary interpretations of findings as statistically significant or not.

This review also has several limitations, similar to the study by Goodman et al. [18]. First, the high heterogeneity in study designs and methodologies restricts the ability to aggregate the results. Therefore, a narrative synthesis instead of a meta-analysis was conducted to analyse the results. While narrative synthesis offers flexibility for complex data [24], it cannot quantitatively weight studies with large samples or a low SE. Second, using the White or White British ethnic category as a fixed benchmark in research can unintentionally suggest that their experiences are the standard [18, 42], potentially reinforcing stereotypes. Moreover, treating the entire White group as homogeneous overlooks important internal variations [18], as the broad category of ‘White’ includes diverse groups such as Irish, White Other, and Gypsy or Irish Traveller, each of which has distinct mental health outcomes [25]. Third, most studies included in the review are community-based and thus lack a clinical-based sample. This kind of approach and sample may not accurately reflect the patterns of diagnosis observed in clinical practice [45]. Fourth, this review predominantly addressed MHPs as identified by the SDQ rather than the clinical diagnoses that the children received (e.g. anxiety, depression, and bipolar disorder). Such reliance on SDQ-defined MHPs may limit the breadth of represented mental health disorders, potentially resulting in an incomplete picture of the varied and clinically significant mental health challenges in children across ethnic groups. Finally, studies that meet the inclusion criteria might have been missed. Despite the adoption of multiple search approaches, ethnicity-related findings are often hidden within the main body of a publication instead of in its abstract, which can make it challenging to include all the studies meeting the inclusion criteria.

## Conclusion

In summary, this systematic review finds that, despite facing more racism and socioeconomic disadvantage [14], ethnic minority children in the UK generally have similar, sometimes lower, mental health risks compared to White British children. The comprehensive analysis using both unadjusted and adjusted models suggests that age, gender, and income might influence mental health risks more than ethnicity. However, the focus on community samples and SDQ-defined MHPs calls for future research using clinical diagnoses and large-scale clinic-based studies for additional understanding of child mental health across ethnic groups in the UK.

## Electronic supplementary material

Below is the link to the electronic supplementary material.

Supplementary Material 1

Supplementary Material 2

Supplementary Material 3

Supplementary Material 4

Supplementary Material 5

## Data Availability

All data generated or analysed during this study are included in this published article and its supplementary information files.
