# Supplementary material for: Associations between ethnicity and mental health problems among children and adolescents in the United Kingdom: A systematic review and narrative synthesis
Source: BMC Public Health. 2024 Nov 25;24:3267. doi: 10.1186/s12889-024-20695-3 (PMC11587580; doi:10.1186/s12889-024-20695-3)
Supplement: Supplementary file 1 — Supplementary Material 1 [file 12889_2024_20695_MOESM1_ESM.docx]

**Additional file 1**

**Electronic search sources**

**Databases:**

Medline, APA PsycINFO, Embase, PubMed, Web of Science, Scopus, and ASSIA

**Web search:**

DuckDuckGo, Million Short, Semantic Scholar, Google scholar, BASE (Bielefeld Academic Search Engine), National Grey Literature Collection, EThOS, Networked Digital Library of Theses and Dissertations, Office for National Statistics, SIREN (Social Interventions Research & Evaluation Network) Evidence & Resource Library, NHS knowledge and library services, The Department of Health National electronic library for health, The King’s Fund, Mind (National Association for Mental Health), NHS digital, Clinical Record Interactive Record (CRIS) publications

**Search terms for each database**

Year restriction: January 2012 - December 2022

Language restriction: English

Search restriction: title and abstract search

| **#** | **Domain** |  | **Search terms** | **Medline** | **Embase** | **APA PsycINFO** | **PubMed** | **Web of Science** | **Scopus** | **ASSIA** |
| --- | --- | --- | --- | --- | --- | --- | --- | --- | --- | --- |
| 1 | Population of interested age groups |  | Child* |  |  |  |  |  |  |  |
|  |  |  | Adolescen* |  |  |  |  |  |  |  |
|  |  |  | Teen* |  |  |  |  |  |  |  |
|  |  |  | Youth* |  |  |  |  |  |  |  |
|  |  |  | Juvenile* |  |  |  |  |  |  |  |
|  |  |  | young people |  |  |  |  |  |  |  |
|  |  |  | young person |  |  |  |  |  |  |  |
|  |  |  | young individual |  |  |  |  |  |  |  |
|  |  |  | young population |  |  |  |  |  |  |  |
|  |  |  | Youngster* |  |  |  |  |  |  |  |
|  |  |  | Schoolchild* |  |  |  |  |  |  |  |
|  |  |  | Preschool* |  |  |  |  |  |  |  |
|  |  |  | Pre-school* |  |  |  |  |  |  |  |
|  |  |  | Minor*1 |  |  |  |  |  |  |  |
|  |  |  | Student* |  |  |  |  |  |  |  |
|  |  |  | Boy* |  |  |  |  |  |  |  |
|  |  |  | Girl* |  |  |  |  |  |  |  |
|  |  |  | exp Adolescent/ |  |  | - |  | - | - |  |
|  |  |  | or exp Child/ |  |  | - |  | - | - |  |
|  |  |  | = |  |  |  |  |  |  |  |
| 2 | Population of interested ethnicity |  | Ethnic* |  |  |  |  |  |  |  |
|  |  |  | Multi-ethnic* |  |  |  |  |  |  |  |
|  |  |  | Multi?ethnic* |  |  |  |  |  |  |  |
|  |  |  | Race* |  |  |  |  |  |  |  |
|  |  |  | Racial* |  |  |  |  |  |  |  |
|  |  |  | Multi-racial* |  |  |  |  |  |  |  |
|  |  |  | Multi?racial* |  |  |  |  |  |  |  |
|  |  |  | Minorit* |  |  |  |  |  |  |  |
|  |  |  | Immigra* |  |  |  |  |  |  |  |
|  |  |  | Migrant* |  |  |  |  |  |  |  |
|  |  |  | Cross?cultural* |  |  |  |  |  |  |  |
|  |  |  | Cross-cultural* |  |  |  |  |  |  |  |
|  |  |  | multi?cultural* |  |  |  |  |  |  |  |
|  |  |  | multi-cultural* |  |  |  |  |  |  |  |
|  |  |  | BAME |  |  |  |  |  |  |  |
|  |  |  | “Black Asian and Minority Ethnic” |  |  |  |  |  |  |  |
|  |  |  | Asian* |  |  |  |  |  |  |  |
|  |  |  | Indian* |  |  |  |  |  |  |  |
|  |  |  | Pakistani* |  |  |  |  |  |  |  |
|  |  |  | Chinese* |  |  |  |  |  |  |  |
|  |  |  | Bangladeshi* |  |  |  |  |  |  |  |
|  |  |  | Black* |  |  |  |  |  |  |  |
|  |  |  | Caribbean* |  |  |  |  |  |  |  |
|  |  |  | African* |  |  |  |  |  |  |  |
|  |  |  | Mixed-race* |  |  |  |  |  |  |  |
|  |  |  | Mixed?race* |  |  |  |  |  |  |  |
|  |  |  | English* |  |  |  |  |  |  |  |
|  |  |  | British* |  |  |  |  |  |  |  |
|  |  |  | Welsh |  |  |  |  |  |  |  |
|  |  |  | Scottish |  |  |  |  |  |  |  |
|  |  |  | “Northern Irish” |  |  |  |  |  |  |  |
|  |  |  | Irish |  |  |  |  |  |  |  |
|  |  |  | Irish adj2 trave?ler* |  |  |  |  |  |  |  |
|  |  |  | exp "Ethnic and Racial Minorities"/ |  |  |  |  | - | - |  |
|  |  |  | Exp Minority Groups/ |  |  |  |  | - | - |  |
|  |  |  | Exp Ethnicity/ |  |  |  |  | - | - |  |
|  |  |  | exp Human Migration/ |  |  |  |  | - | - |  |
|  |  |  | = |  |  |  |  |  |  |  |
| 3 | Location |  | UK |  |  |  |  |  |  |  |
|  |  |  | “United Kingdom” |  |  |  |  |  |  |  |
|  |  |  | Britain |  |  |  |  |  |  |  |
|  |  |  | England |  |  |  |  |  |  |  |
|  |  |  | Scotland |  |  |  |  |  |  |  |
|  |  |  | Wales |  |  |  |  |  |  |  |
|  |  |  | “Northern Ireland” |  |  |  |  |  |  |  |
|  |  |  | exp United Kingdom/ |  |  |  |  | - | - |  |
|  |  |  | = |  |  |  |  |  |  |  |
| 4 | Mental Health Outcomes |  | “Mental health” |  |  |  |  |  |  |  |
|  |  |  | exp Mental Health/ |  |  |  |  | - | - |  |
|  |  |  | Mental ill-health |  |  |  |  |  |  |  |
|  |  |  | exp Mental Disorders/ |  |  |  |  | - | - |  |
|  |  |  | Psycho* adj2 (disor* OR ill* OR problem* OR symptom*) |  |  |  |  |  |  |  |
|  |  |  | Mental adj2 (disor* OR ill* OR problem* OR symptom*) |  |  |  |  |  |  |  |
|  |  |  | Psycho* |  |  |  |  |  |  |  |
|  |  |  | Psychiatric |  |  |  |  |  |  |  |
|  |  |  | Depress* |  |  |  |  |  |  |  |
|  |  |  | Anxi* |  |  |  |  |  |  |  |
|  |  |  | Internali* |  |  |  |  |  |  |  |
|  |  |  | Externali* |  |  |  |  |  |  |  |
|  |  |  | Conduct adj2 (disor* OR ill* OR problem* OR symptom*) |  |  |  |  |  |  |  |
|  |  |  | PTSD |  |  |  |  |  |  |  |
|  |  |  | Post-traumatic stress disorder |  |  |  |  |  |  |  |
|  |  |  | Eating adj2 disorder* |  |  |  |  |  |  |  |
|  |  |  | anorexia |  |  |  |  |  |  |  |
|  |  |  | bulimia |  |  |  |  |  |  |  |
|  |  |  | Emotion* adj2 (disorder* OR ill* OR problem* OR symptom*) |  |  |  |  |  |  |  |
|  |  |  | Behavio?r* adj2 (disorder* OR ill* OR problem* OR symptom*) |  |  |  |  |  |  |  |
|  |  |  | Tic adj2 disorder* |  |  |  |  |  |  |  |
|  |  |  | ODD |  |  |  |  |  |  |  |
|  |  |  | Oppositional adj2 defiant |  |  |  |  |  |  |  |
|  |  |  | Self-harm |  |  |  |  |  |  |  |
|  |  |  | Self-injure |  |  |  |  |  |  |  |
|  |  |  | “self‐injurious behavior” |  |  |  |  |  |  |  |
|  |  |  | Suicid* |  |  |  |  |  |  |  |
|  |  |  | OCD |  |  |  |  |  |  |  |
|  |  |  | Obsessive adj2 compulsive |  |  |  |  |  |  |  |
|  |  |  | Schizophrenia |  |  |  |  |  |  |  |
|  |  |  | DE "Chronic Mental Illness" OR DE "Chronic Psychosis" OR DE "Serious Mental Illness" OR DE "Mental Illness (Attitudes Toward)" OR DE "Illness Anxiety Disorder" | - | - |  | - | - | - | - |
|  |  |  | = |  |  |  |  |  |  |  |
| 5 | Mental health services |  | CAMHS |  |  |  |  |  |  |  |
|  |  |  | “Child and adolescent mental health services” |  |  |  |  |  |  |  |
|  |  |  | mental adj service* |  |  |  |  |  |  |  |
|  |  |  | Mental health care |  |  |  |  |  |  |  |
|  |  |  | Psychiatr* adj service* |  |  |  |  |  |  |  |
|  |  |  | Psycholog* adj2 specialist* |  |  |  |  |  |  |  |
|  |  |  | psycho* adj2 clinic* |  |  |  |  |  |  |  |
|  |  |  | Psychiatrist* |  |  |  |  |  |  |  |
|  |  |  | Psychologist* |  |  |  |  |  |  |  |
|  |  |  | “Psycholog* therapist*” |  |  |  |  |  |  |  |
|  |  |  | exp Mental Health Services/ |  |  |  |  | - | - |  |
|  |  |  | Exp Child Health Services/ |  |  | - |  | - | - |  |
|  |  |  | Exp Adolescent Health Services/ |  |  | - |  | - | - |  |
|  |  |  | DE “School Based Mental Health Services” | - | - |  | - | - | - | - |
|  |  |  | = |  |  |  |  |  |  |  |
| 6 | Combine |  | #1 AND #2 AND #3 AND #4 AND #5 |  |  |  |  |  |  |  |
