# Supplementary material for: Associations between ethnicity and mental health problems among children and adolescents in the United Kingdom: A systematic review and narrative synthesis
Source: BMC Public Health. 2024 Nov 25;24:3267. doi: 10.1186/s12889-024-20695-3 (PMC11587580; doi:10.1186/s12889-024-20695-3)
Supplement: Supplementary file 4 — Supplementary Material 4 [file 12889_2024_20695_MOESM4_ESM.docx]

**Quality assessment criteria**

**Individual question’s quality rating:**

**1. Did the study address a clearly focused issue?**

High quality:

Clearly answer the research questions.

A clear and precise description of the population, categories of ethnic groups and types of mental health outcomes.

Moderate quality:

Answer the research questions but not in-depth.

There are ethnic groups but vague assignment methods.

There are mental health outcomes, but the definitions are vague.

Low quality: neither ethnic groups nor mental health outcomes are clearly defined.

**2. Was the cohort recruited in an acceptable way?**

High quality: clear representation of ethnic backgrounds, clear description, and justification for participant selection.

Moderate quality: some ethnic backgrounds were represented, and some justification was provided.

Low quality: selective representation of ethnic backgrounds, no clear justification.

**3. Was the exposure accurately measured to minimise bias?**

High quality: validated and evidence-based measurements or UK census were used to assign ethnic groups. All the participants were classified into exposure groups using the same procedure. Please note that if the study lacks a specific description of the measurement but uses a widely used dataset (e.g., MCS) that is considered reliable, it is still considered ‘high quality’.

Moderate quality: self-report ethnic groups without using validated and evidence-based measurements or the UK census.

Low quality: ambiguous or unclear methods to determine ethnicity.

**4. Was the outcome accurately measured to minimise bias?**

High quality: use of validated mental health measurement methods that can be used for children and adolescents. For example, a description of the use of specific SDQ subscales, or a clearly described standardised diagnostic process or criteria used by GPs or specialists. Please note that if the study lacks a specific description of the measurement but uses a widely used dataset (e.g., MCS) that is considered reliable, it is still considered ‘high quality’.

Moderate quality: use of validated mental health assessment tools without a description of the specific subscales or diagnostic criteria adopted by GPs or specialists.

Low quality: ambiguous or non-standardised/validated assessments.

**5. (a) have the authors identified all important confounding factors?**

High quality: comprehensive list of potentially important confounders considered.

Moderate quality: some confounders were identified but not exhaustive.

Low quality: minimal to no confounders identified.

**5. (b) have they taken account of the confounding factors in the design and/or analysis?**

High quality: use of statistical methods to take into account all identified confounders.

Moderate quality: some statistical controls were used, but not for all identified confounders.

Low quality: no statistical method was used to control for confounders.

**6. Was the follow up of subjects (a) complete enough and (b) long enough?**

(given the primary importance of exploring the association, this question is removed from the checklist for this review)

**Section b: what are the results?**

**7. What are the results of this study?** (Focus: how the study presents its findings)

High quality: the association between specific ethnicity and mental health outcomes should be clearly indicated with a statistical measure like or, RR, etc. If confidence intervals and p-values are provided, that further strengthens the quality of the result presentation, as they indicate the precision and significance of the findings.

*(Please note that the focus should be on the results of the associations, but also needs to consider other results indicated in the overall contexts)*

Moderate quality: results are reported in a way that is generally clear but may have certain ambiguities. For example, 1) authors might use high-level ethnicity assignment when they look at sub-ethnic-groups, or they might use a general or umbrella term to describe mental disorders when they look at specific disorders (e.g., ‘emotional disorders’ without specifying if it's depression, anxiety, etc.), although the aim of that study is to explore associations between ethnic groups and particular disorder.

*(Note: most included studies did not specify the type of mental health problem, this might be a limitation in my systematic review. The use of general or umbrella terms might limit the ability to draw specific conclusions about specific disorders, but it could provide an overview of overall mental health problems among the populations studied.)*

2) another example could be that they mention an association but do not provide specific statistical measures like or, making it harder to understand the strength or significance of the association.

Low quality: ambiguous presentation of results.

**8. How precise are the results?**

High quality: provides narrow confidence intervals or other measures of precision.

Moderate quality: provides confidence intervals but some were with wide ranges.

Low quality: no measures of precision

**9. Do you believe the results?**

High quality:

Methodology effectively minimises biases, chance, and confounders. Seldom or no methodology flaws (e.g., too small sample size, no controlling for key confounders). The high precision of results was shown by narrow confidence intervals.

*(Please note that the hints next to this question in the checklist - time sequence and dose-response gradient - are not necessarily observed for this systematic review. This is because 1) ethnicity is an inherent characteristic that has no clear starting point, unlike a drug intervention. 2) mental health problems can be either genetical or social-driven. 3) the included studies are either cross-sectional (time-irrelevant) or longitudinal (understanding the progression of mental health problems across ethnicities).)*

Moderate quality:

Some potential biases or confounders may not have been fully addressed. The precision of results was moderate. For example, a study finds a relationship between ethnicity and mental health but only sampled from low-income neighbourhoods, it might be confounded by socio-economic status. If not addressed, this would be a limitation.

Low quality:

Major methodological flaws are evident, making results potentially unreliable.

**Section c: will the results help locally?**

**10. Can the results be applied to the local population?**

High quality: highly reflective of the broader UK context for ethnic diversity, which supports generalisability.

Moderate quality: some reflection of the UK context but may have limitations. The study reflects some aspects of the UK's ethnic diversity but may not be entirely representative. Obvious differences exist between the study and local settings, but they are not too vast to invalidate findings.

Low quality: the results cannot be generalised to the broader UK context.

**11. Do the results of this study fit with other available evidence?**

High quality: results align with existing literature or provide valid and robust reasons for discrepancies (e.g., opposite findings or highly unexpected findings like no association observed).

Moderate quality: results partially align with previous literature and provide a reasonable discussion of why their results might differ. But may lack in-depth and comprehensive justifications (i.e., superficial explanations).

Low quality: results are totally different from previous literature without reasonable justifications.

**12. What are the implications of this study for practice?**

High quality: robust implications for practice. The study’s recommendations are presented as part of a more conclusive body of evidence. While the study acknowledges the limitations of observational research, its recommendations are based on thorough analysis and are supported by a broader body of evidence.

Moderate quality: preliminary implications for practice. Might lack clarification on how the findings should be translated into practice or policy.

Low quality: weak or unclear implications for practice. The study provides implications for practice that are either too broad, vague, or not well-supported by its results.

Limited acknowledgement of the inherent limitations of observational studies (e.g., no causation, recall bias, loss to follow-up etc.), leading to possibly overstated recommendations. Few or no existing evidence to judge the study's recommendations.

**Overall quality rating:**

**High quality study:**

Meets "high quality" criteria on most of the checklist questions.

Contains minimal methodological drawbacks that could bias results.

Provides clear and detailed descriptions of methodology, which can assess mental health in a valid way.

High quality of ethnic groups assignment.

The study provides reliable data and relevant interpretation for associations between ethnicity and mental health problems among children and adolescents in the UK.

**Moderate quality study:**

Meets "high quality" criteria on some checklist questions, but also "moderate quality" or possibly "low quality" on others.

Contains some methodological drawbacks that might introduce bias, but these are acknowledged by the authors or can be recognized in the context of the broader literature.

The study provides valuable insights, but still need to be careful when interpreting its results due to the limitations.

**Low quality study:**

Meets "low quality" criteria on many checklist questions.

Contains various methodological drawbacks or lacks sufficient detail of the methodology, making it challenging to fully trust the findings.

The study's findings should be interpreted with caution and additional evidence from more robust studies that may be needed before drawing strong conclusions.
